# The Exposition—Farewell

**Published:** 1901-11

**Authors:** 


### The Exposition—Farewell.

THE closing days of the great Pan-American Fair have been reached, and while these lines are writing it is announced that the last day will be November 2. The director-general, who goes to Mexico as a delegate to the Pan-American Congress, has taken his leave, and all the officials are making preparations for the closure of one of the most interesting exhibitions ever held on this continent.

We have not time nor inclination now to deal with the fair in all its important relations to the community, nor in criticism of its management, which latter has been a subject of both praise and blame, but there are one or two features that relate to medicine which we desire to recall. It is to the great credit of the medical director, Dr. Roswell Park, that he insisted upon the construction of a proper hospital,—a triumph which was obtained over much opposition, we believe. The wisdom of building the hospital was over and again demonstrated, but especially in the case of President McKinley, where the operation on him was made within two hours after the wounds were inflicted.

Again, the thorough sanitation of the fair is a subject of medical pride. Not one serious inconvenience has been experienced by reason of any unsanitary conditions of the grounds, but on the other hand, often at much cost of time and patience, there has been most complete inspection and insistence of thorough cleanliness by the sanitary officer, and for this the thanks of the community are due to the incessant labors of Dr. Nelson W. Wilson.

We should like to mention other matters of interest, but must defer until another time a general commentary on the Rainbow City, contenting ourselves now with these two important allusions to the conduct of the fair and a mere regretful—Farewell.

